# MV-Flow and LumiFlow: new Doppler tools for the visualization of fetal blood vessels

**DOI:** 10.1590/0100-3984.2020.0109

**Published:** 2021

**Authors:** André de Souza Malho, Renato Ximenes, Adriana Ferri, Nathalie Jeanne Bravo-Valenzuela, Edward Araujo Júnior

**Affiliations:** 1 Latin American Fetal Medicine Foundation (FMF-LA), Campinas, SP, Brazil.; 2 Sector of Fetal Medicine, Hospital e Maternidade Santa Joana, São Paulo, SP, Brazil.; 3 Ultrasound Clinical Education, Samsung Healthcare Brazil, São Paulo, SP, Brazil.; 4 Discipline of Pediatrics (Pediatric Cardiology), Department of Internal Medicine, Universidade Federal do Rio de Janeiro (UFRJ), Rio de Janeiro, RJ, Brazil.; 5 Department of Obstetrics, Escola Paulista de Medicina da Universidade Federal de São Paulo (EPM-Unifesp), São Paulo, SP, Brazil.; 6 Medical Course, Universidade Municipal de São Caetano do Sul/Campus Bela Vista, São Paulo, SP, Brazil.

The advanced power Doppler technique known as MV-Flow (the MV standing for microvascular) is a new tool to detect microvascular flow that cannot be visualized on conventional Doppler images. Because this new tool can show the blood flow in areas in which it was previously undetectable, we anticipate that it will improve diagnostic reliability. Increased usage of this tool is expected to provide many clinical benefits. In conventional color Doppler or power Doppler, very low-velocity blood flow are detected within the same velocity range as the tissue^(1,2)^, causing the ultrasound system to recognize the low-velocity blood flow as noise and remove them. Therefore, blood flow at a velocity below the constant (< 200 Hz) cannot be detected with those techniques. However, MV-Flow offers a detailed view of low-velocity blood flow in relation to the surrounding tissues, with high sensitivity and resolution. Thus, unlike conventional Doppler, MV-Flow can detect microvascular (microfluidic channel) flow into tissues and organs.

LumiFlow is an advanced post-processing and shading tool. A typical color image is two-dimensional and therefore looks flat. However, these images can be processed by placing a light source on the right, which generates shadows and creates a three-dimensional (3D) effect. A typical color image is not made of just a single color. The blood flow at the center of the vessel has the highest velocity, which is expressed as a lighter color, whereas blood flow at the vessel boundaries, which has the lowest velocity, is expressed as a darker color. Likewise, on LumiFlow imaging, higher velocities and reflections are brighter, whereas lower velocities and shadows are darker. LumiFlow is a post-processing tool that allows the display of existing color or power Doppler images and provides 3D visualization of the vascular image in real time, as does MV-Flow. One advantage of LumiFlow is that it makes the flow boundaries appear relatively darker, creating clearer distinctions between blood vessels and providing better removal of background noise.

To our knowledge, there has been only one study analyzing MV-Flow and LumiFlow in the evaluation of the fetal microvasculature. Dall’Asta et al.^(3)^ assessed the vascularization of the torcular herophili (TH) in the second trimester of pregnancy. The authors evaluated the anatomical relationship between the TH and the “transpalatal line” that connects the hard palate to the skull. They reported that, in normal fetuses, the TH appeared to lie on or just below that line. In cases of Blake’s pouch cyst, the authors found that the TH appeared in a normal position (i.e., the same as that observed in the controls), whereas the TH was highly elevated in relation to the transpalatal line in cases of Dandy-Walker malformation. In a fetus with Chiari II malformation, they found that the TH was below the line.

**Figure 1:**
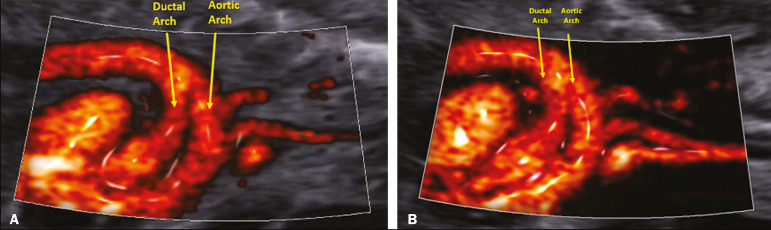
Sagittal view of a fetal heart with TGA at 18 weeks and 3 days showing the aortic and ductal arches by MV-Flow and LumiFlow (**A**), as well as by twodimensional digital subtraction (**B**).

**Figure 2:**
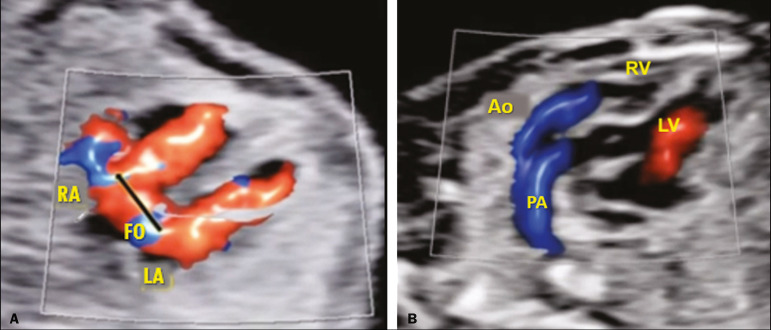
Four-chamber view of a fetal heart with TGA at 18 weeks and 3 days showing a patent foramen ovale (**A**). The outflow tract shows two parallel vessels by S-flow (directional power) with LumiFlow (**B**). RA, right atrium; FO, foramen ovale; LA, left atrium; Ao, aorta; PA, pulmonary artery; RV, right ventricle; LV, left ventricle.

To demonstrate the advantages of the MV-Flow and LumiFlow tools, we assessed a 39-year-old pregnant woman (first pregnancy) and her fetus, at 18 weeks and 4 days of gestation, with a diagnosis of simple transposition of the great arteries (TGA), using the HERA W10 ultrasound system (Samsung Co., Seoul, South Korea). Simple TGA is a congenital heart disease that is still underdiagnosed *in utero*, with a detection rate below 50%. The prenatal diagnosis of TGA is crucial to improving postnatal outcomes^(4)^. Classically, the diagnosis of TGA is based on the identification of the origin of each of the great arteries. In this setting, we used the MV-Flow and LumiFlow tools to perform a detailed analyses of the outflow tracts and determine the diagnosis. Although MV-Flow is typically used in the assessment of the adult microvasculature, it can be applied to the fetal heart because it has a high frame rate. We made adjustments in MV-Flow and LumiFlow to improve the visualization of the aortic and ductal arches. LumiFlow was able to simulate a 3D view of the fetal heart structures (Figure 1). Because this technique shows the vessel margins in a darker color, it allowed us to differentiate between different fetal heart vessels. In addition, LumiFlow may be used in combination with other modes such as color Doppler, S-Flow, and power Doppler. The darker margin of the fetal heart vessels shows the similarity of 3D vision (Figure 2). To the best of our knowledge, there are no previous reports of using MV-Flow and LumiFlow in concert to assess fetal vessels.

In summary, MV-Flow and LumiFlow may improve the diagnosis of vascular anomalies in the fetal heart, especially when detailed assessments of the great arteries are required. MV-Flow and LumiFlow are both advanced tools that facilitate understanding of vessel boundaries, rapidly providing additional spatial information regarding venous and arterial anomalies.
